# *SCN8A* mutation in a child presenting with seizures and developmental delays

**DOI:** 10.1101/mcs.a001073

**Published:** 2016-11

**Authors:** Janet Malcolmson, Robert Kleyner, David Tegay, Whit Adams, Kenneth Ward, Justine Coppinger, Lesa Nelson, Miriam H. Meisler, Kai Wang, Reid Robison, Gholson J. Lyon

**Affiliations:** 1 Stanley Institute for Cognitive Genomics, Cold Spring Harbor Laboratory, Cold Spring Harbor, New York 11724, USA;; 2 Genetic Counseling Graduate Program, Long Island University (LIU), Brookville, New York 11548, USA;; 3 Utah Foundation for Biomedical Research, Salt Lake City, Utah 84107, USA;; 4 Affiliated Genetics, Salt Lake City, Utah 84109, USA;; 5 Department of Human Genetics, University of Michigan, Ann Arbor, Michigan 48109-5618, USA;; 6 Zilkha Neurogenetic Institute, University of Southern California, Los Angeles, California 90089, USA;; 7 Department of Psychiatry and Behavioral Sciences, Keck School of Medicine, University of Southern California, Los Angeles, California 90033, USA

**Keywords:** absent speech, action tremor, appendicular hypotonia, blepharospasm, broad forehead, bulbar palsy, developmental regression, developmental stagnation at onset of seizures, dysphagia, epileptic encephalopathy, exaggerated startle response, failure to thrive in infancy, full cheeks, gastroesophageal reflux, gastrostomy tube feeding in infancy, generalized tonic seizures, generalized tonic-clonic seizures on awakening, gingival overgrowth, hypoxemia, infantile axial hypotonia, intellectual disability, severe, neck muscle weakness, no social interaction, obstructive sleep apnea, respiratory difficulties

## Abstract

The *SCN8A* gene encodes the sodium voltage-gated channel alpha subunit 8. Mutations in this gene have been associated with early infantile epileptic encephalopathy type 13. With the use of whole-exome sequencing, a de novo missense mutation in *SCN8A* was identified in a 4-yr-old female who initially exhibited symptoms of epilepsy at the age of 5 mo that progressed to a severe condition with very little movement, including being unable to sit or walk on her own.

## INTRODUCTION

Mutations in *SCN8A* are associated with cognitive impairment with or without cerebellar ataxia (OMIM#613406) and with early infantile epileptic encephalopathy-13 (EIEE13, OMIM#614558). Loss-of-function mutations can be associated with cerebellar ataxia and cognitive issues, whereas gain-of-function mutations can underlie epileptic encephalopathy (O'Brien and Meisler 2013). De novo mutations in *SCN8A* have been discovered through genome and exome sequencing, and *SCN8A* can now be evaluated with commercial epilepsy panels.

Epileptic encephalopathy is characterized by seizure activity that progresses to cerebral dysfunction leading to severe cognitive, motor, and behavioral impairments (Vaher et al. 2014). Approximately 1% of early infantile epileptic encephalopathies are associated with missense mutations in the *SCN8A* gene, and approximately 50 cases have been described in the literature (Veeramah et al. 2012; Larsen et al. 2015; Wagnon and Meisler 2015; Meisler et al. 2016). In a few cases, the mutation was inherited from a mosaic parent, but the majority of disease-contributory mutations are de novo missense mutations (Wagnon and Meisler 2015). EIEE13 has an average age of onset between 3 and 7 mo, and individuals present with various types of seizures, including tonic–clonic, generalized tonic, atonic, myoclonic, and focal and absence seizures, whereas febrile seizures are rare (Ohba et al. 2014). There is often developmental regression, and movement disorders are present with 50% of affected individuals unable to sit or walk (Meisler et al. 2016).

*SCN8A* is located on Chromosome 12q13 and encodes the sodium voltage-gated channel alpha subunit (also know as Na_v_1.6), which functions in the rapid depolarization of sodium channels during generation of action potentials in neurons. Na_v_1.6 is one of the three major sodium channels in the brain and is involved in the regulation and propagation of firing patterns of excitatory and inhibitory neurons (O'Brien and Meisler 2013). Na_v_1.6 is localized to the axonal initial segment and to nodes of Ranvier in myelinated neurons. Mutations in the related sodium channel gene *SCN1A* contribute to Dravet syndrome, and mutations in *SCN2A* contribute to Ohtahara syndrome (Wagnon and Meisler 2015).

Ten patient mutations of *SCN8A* have been evaluated with functional tests in transfected cells (Veeramah et al. 2012; de Kovel et al. 2014; Estacion et al. 2014; Blanchard et al. 2015; Wagnon et al. 2016). In eight of the 10 cases, gain-of-function effects were observed. The most common defect is impaired channel inactivation, which leads to a persistent sodium current and increased neuronal activity. In two cases, a hyperpolarizing shift in voltage dependence of channel activation was observed, also leading to hyperactivity. This gain-of-function mechanism is opposite to the situation in Dravet syndrome, where loss-of-function mutations in *SCN1A* are most common (Meisler et al. 2016).

Through advances in DNA sequencing technology, DNA can now be sequenced quickly and relatively inexpensively. Although such analyses focus on ∼1% of the human genome, this, nonetheless, still identifies tens of thousands of variants to analyze (O'Rawe et al. 2013, 2015; Lyon and O'Rawe 2015). Through the use of existing and novel bioinformatics tools, we developed a protocol focused on identifying uncommon and deleterious mutations and prioritizing them by phenotypic relevance. We present here a detailed case study of a proband in which a de novo likely disease-contributing mutation in *SCN8A* was discovered. This study focused on the use of next-generation exome sequencing to find mutations by analyzing primarily protein-coding regions of the DNA.

## RESULTS

### Clinical Presentation and Family History

**Figure 1.**
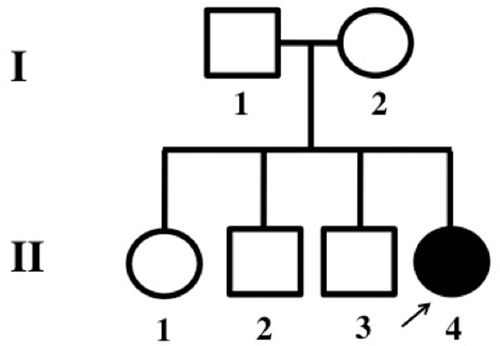
Pedigree: II-4, the affected proband (4-yr-old), is the daughter of an unaffected, nonconsanguineous couple. The proband has one older (14-yr-old) unaffected sister and two older (11-yr-old and 7-yr-old) unaffected brothers.

**Figure 2.**
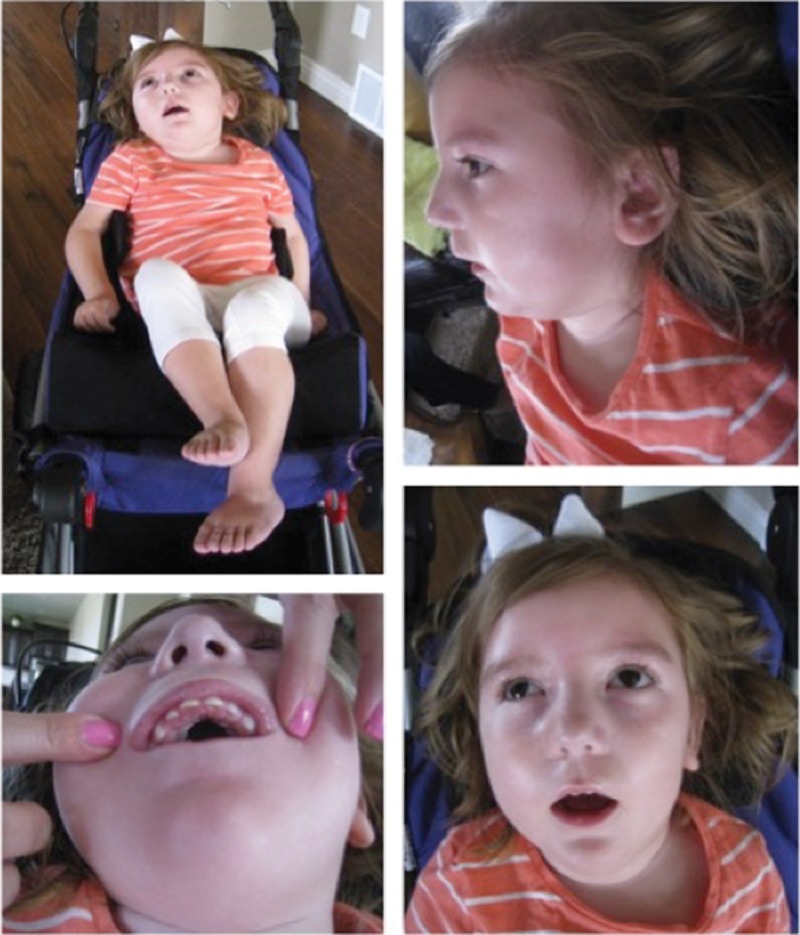
Pictures of phenotype of proband. Facial features include brachycephaly, broad forehead, broad nasal root, hypoplastic alae nasi, full cheeks, gingival hyperplasia, mild micrognathia, and hypotonic facies.

The proband is a 4-yr-old female presenting with idiopathic epilepsy (10 to 15 seizures per day), cortical blindness, and developmental regression (Figs. 1 and 2; Supplemental Video 1). Her medical records show that she has no language or motor skills, is fed through a G tube, and has recurrent fevers and osteopenia. She had acute fractures of her distal radius and distal ulna bilaterally as well as on the left distal tibia and left distal fibula while on a ketogenic diet. Table 1 outlines her phenotypic features using Human Phenotype Ontology (HPO) terms. Before whole-exome sequencing (WES), an infantile epileptic encephalopathy panel that did not include *SCN8A* was ordered for this proband. The results showed a variant of uncertain significance in exon 3 of the *GRIN2A* gene that encodes the glutamate ionotropic receptor *N*-methyl-d-aspartate (NMDA)-type subunit 2A, resulting in an amino acid change from phenylalanine to isoleucine at position 183. Parents of the proband were tested for this variant and the unaffected father was found to carry this variant. This proband had a high-resolution whole-genome single-nucleotide polymorphism (SNP)/copy-number microarray analysis using the Cytoscan HD platform, and Chromosome Analysis Suite Software (Affymetrix) was used for analysis. The result was normal and no clinically significant copy-number variants were discovered.

**Table 1.** Summary of the clinical features in this proband

Features (Human Phenotype Ontology ID)	Proband
Epilepsy	
Epileptic encephalopathy (HP:0200134)	+
Generalized tonic seizures (HP:0010818)	+
Generalized tonic-clonic seizures on awakening (HP:0007193)	+
EEG abnormality (HP:0002353)	+
Developmental/intellectual disability	
Global developmental delay (HP:0001263)	+
Developmental stagnation at onset of seizures (HP:0006834)	+
Developmental regression (HP:0002376)	+
Absent speech (HP:0001344)	+
Intellectual disability, severe (HP:0010864)	+
Motor delay (HP:0001270)	+
Neurological	
Generalized hypotonia (HP:0001290)	+
Appendicular hypotonia (HP:0012389)	+
Infantile axial hypotonia (HP:0009062)	+
Neck muscle weakness (HP:0000467)	+
Rigidity (HP:0002063)	+
Spasticity (HP:0001257)	+
Growth/feeding	
Failure to thrive in infancy (HP:0001531)	+
Gastrostomy tube feeding in infancy (HP:0011471)	+
Gastroesophageal reflux (HP:0002020)	+
Dysphagia (HP:0002015)	+
Respiratory	
Respiratory difficulties (HP:0002880)	+
Hypoxemia (HP:0012418)	+
Abnormality of the tonsils (HP:0100765)	+
Obstructive sleep apnea (HP:0002870)	+
Dysmorphism	
Brachycephaly (HP:0000248)	+
Broad forehead (HP:0000337)	+
Broad nasal root or bridge (HP:0000431)	+
Hypoplastic alae nasi (HP:0000430)	+
Full cheeks (HP:0000293)	+
Gingival overgrowth (HP:0000212)	+
Micrognathia (mild) (HP:0000347)	+
Hypotonic facies	+
Neurological	
Exaggerated startle response (HP:0002267)	+
Action tremor (HP:0002345)	+
Blepharospasm (HP:0000643)	+
Bulbar palsy (HP:0001283)	+
Nystagmus (HP:0000639)	+
Miscellaneous	
Hyperreflexia (HP:0001347)	+
No social interaction (HP:0008763)	+

EEG, electroencephalograph.

The only reported complication during pregnancy was a group B streptococcal infection in the mother. From birth to 3 mo of age, the proband met all milestones, although her parents report she was slightly hypotonic. At 3 mo, she experienced her first seizure-like activity, although medical tests revealed no abnormalities. The evening after her 5-mo vaccinations, including diphtheria (DT), *Haemophilus influenzae* type B (Hib) conjugate (PRP-T), inactivated polio vaccine (IPV), and pneumococcal conjugate vaccine (PCV13), she was admitted to the emergency department with increased seizure activity, nystagmus, hypotonia, and fever, although medical tests revealed no pertinent abnormalities. A direct link between the vaccination and the onset of symptoms has not been established, and a link is not proposed by the authors.

An electroencephalogram (EEG) 2 wk after the onset of severe symptoms revealed severe abnormalities, and the patient was diagnosed with idiopathic epileptic encephalopathy. The EEG showed the presence of frequent independent multifocal, mostly right posterior, epileptogenic abnormalities, consistent with a tendency toward partial onset seizures, and this is indicative of right hemispheric profound cerebral dysfunction. Follow-up EEGs showed multifocal sharp waves. Facial features include a short, upturned nose, full cheeks, a short philtrum, and a horizontal crease on the chin (Fig. 2). Magnetic resonance imaging (MRI) scans revealed no significant abnormalities that might contribute to the proband's condition; myelination was determined to be within normal range, and no congenital brain malformations or cytotoxic edema were found to suggest ischemia. The only abnormality found was a small, left periventricular cyst, most likely an incidental germinolytic cyst, which was determined to be unrelated to the proband's clinical presentation. Her physical manifestations include the absence of all mobility and motor coordination, with daily placement in a wheelchair, and the absence of a startle reflex, possibly secondary to neck weakness and poor eye control. The family history is unremarkable.

Medications that the proband is now taking include phenobarbital, carbamazepine, and Charlotte's Web (Hemp extract). The addition of carbamazepine has stopped her loop seizures and prevents her from having seizures as she enters and exits sleep. She has improved awareness, has more eye contact, and is able to shake a rattle.

### Genomic Analysis

Blood samples from the proband, as well as blood and saliva samples from parents and siblings, were sequenced at Affiliated Genetics in Salt Lake City, Utah, where genomic DNA was extracted and exons sequenced using the Life Technologies Ampliseq Exome RDY kit and the Life Technologies Proton sequencing system (see Methods). These targeted regions were sequenced using the Ion Proton sequencing system using Ion Hi-Q Chemistry with 200-bp reads. The DNA sequence was compared with the University of California, Santa Cruz (UCSC) hg19 reference sequence, and several methods of analysis were applied to the sequence data (see Methods). A summary of variants called for all individuals in the family are described in Table 2, and coverage and mapping statistics are shown in Table 3. These analyses included in-house protocols, and several commercial software packages, including Tute Genomics, Omicia Opal, and Cartagenia v4.1, along with the use of the OTG-snpcaller pipeline (see Methods). In all analyses, we arrived at similar conclusions, but the various analyses helped to provide a more comprehensive and in-depth approach to the data. The Omicia results from three “QUAD” analyses (each with different siblings) are shown in Supplemental Tables 1–3. The scripts and programs created or rewritten by the author (R.K.) and used for analysis were uploaded to GitHub and can be found at https://github.com/rkleyner/PGM-WES-Pipeline.

**Table 2.** Count of single-nucleotide polymorphisms (SNPs), insertions and deletions (indels), and the total number of variants for each individual sequenced

Individual	Number of SNPs	Number of indels	Total number of variants
Proband	20,390	1756	22,146
Mother	20,889	1602	22,491
Father	20,705	1764	22,469
Brother 1	21,199	1441	22,640
Brother 2	21,302	1385	22,687
Sister	20,930	1705	22,635

**Table 3.** Average read depth (exonic regions only), number of reads, and percent of reads mapped for each individual sequenced

Individual	Average read depth	Number of reads	Reads mapped (%)
Proband	99.32	36,808,143	98.60
Mother	108.11	39,848,858	98.78
Father	93.46	34,780,797	98.39
Brother 1	85.83	32,467,105	97.47
Brother 2	105.85	41,219,805	96.64
Sister	125.08	45,749,335	98.72

As one example, for the OTG-snpcaller pipeline, the final variant call format (VCF) file for each individual contained 20,000 to 25,000 variants, of which 300 to 400 were found to be inherited. However, 2000 to 3000 variants were recognized as de novo, which is notably above the expected number of de novo mutations found in standard WES (Bamshad et al. 2011; Kong et al. 2012). In addition, even with an optimized variant calling pipeline, there were still a significant number of false positives. The number of variants found in the proband was 28,681 and the mean depth of coverage was 101-fold.

**Figure 3.**
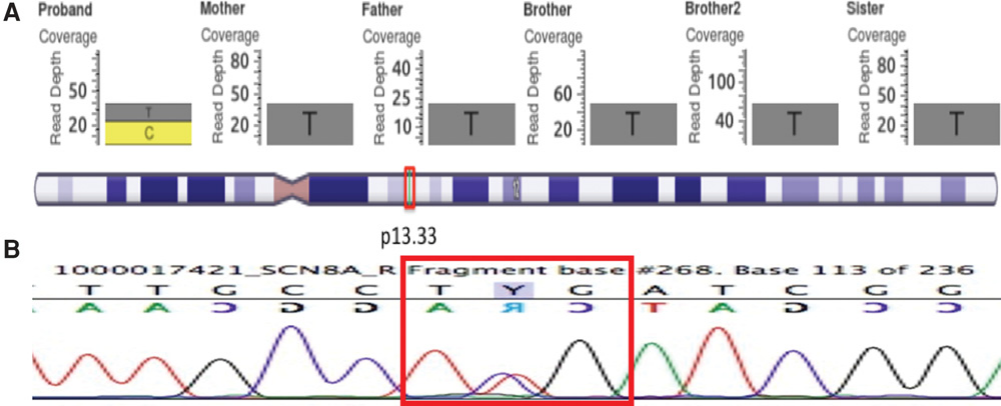
GenomeBrowse output for the mutation in *SCN8A* (*A*). The proband has a heterozygous C substitution in Chromosome 12, position 52,093,447 in *SCN8A.* There are more than 20 reads covering the region, indicating that this is likely a true-positive mutation. None of the other family members appears to have this mutation, categorizing it as de novo. (*B*) Sanger sequencing for the proband confirms the presence of the mutation, as seen by the reverse (*top*) and forward (*bottom*) strands highlighted by the red box.

**Figure 4.**
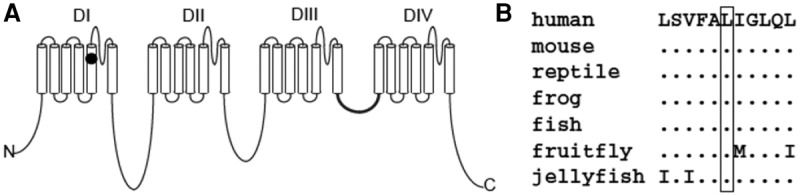
The de novo mutation Leu267Ser in *SCN8A*. Mammals and invertebrate sodium channel genes also have a conserved leucine in this location of the gene. (*A*) The site of the mutation is shown with a black circle. (*B*) This residue is highly conserved during evolution of vertebrate and invertebrate sodium channels.

A GEMINI query (Paila et al. 2013) selected zero autosomal recessive mutations and 16 rare de novo mutations of interest. A unique de novo heterozygous variant in the *SCN8A* gene on Chromosome 12 at position 52,093,447 was identified (c.800T>C, p.Leu267Ser) (Table 4; Fig. 3A). This mutation is not present in any frequency in public databases such as Database for Short Genomic Variations (dbSNP) 137, 1000 Genomes phase 1 data, National Heart, Lung, and Blood Institute (NHLBI) 6500 exomes, or Exome Aggregation Consortium (ExAC) version 0.2, which contains allelic information derived from approximately 60,000 exome sequences. The presence of the mutation was confirmed using Sanger sequencing following standard procedures (Fig. 3B). This missense mutation has not been previously identified in any other patient with epilepsy. Leucine 267 is located in transmembrane segment DIS5 of the sodium channel and is highly conserved through evolution (Fig. 4). The substitution changes the hydrophobic leucine residue to the hydrophilic serine, which is likely to alter channel function. The Combined Annotation-Dependent Depletion (CADD) score was 21.1, indicating that this substitution is within the 0.78% of most deleterious mutations (99.2 percentile). The Sorting Intolerant from Tolerant (SIFT) (Ng and Henikoff 2003) and Polymorphism Phenotyping (PolyPhen) scores (Adzhubei et al. 2013) were <0.01 and >0.999, respectively, also predicting that the mutation is deleterious. Analyses with Phenolyzer (Yang et al. 2015), wANNOVAR (Chang and Wang 2012), and PhenIX (Zemojtel et al. 2014) all predict that a heterozygous missense mutation in *SCN8A* is likely to contribute to the phenotype. The output from PhenIX, Phenolyzer, and wANNOVAR can be viewed in Supplemental Figures 1–3, respectively.

**Table 4.** *SCN8A* variant

Chr:position GRCh37(hg19)	HGVS cDNA	HGVS protein	Type of variant	Predicted effect	Genotype	Parent of origin
12:52,093,447	c.800T>C	p.Leu267Ser	Substitution	Missense	Heterozygous	De novo

HGVS, Human Genome Variation Society.

The Online Mendelian Inheritance in Man (OMIM) database describes early infantile epileptic encephalopathy and cognitive impairment as two phenotypes associated with mutations in *SCN8A*. Because the proband has a similar phenotype, this is considered to be a mutation that contributes to the phenotype described here. Several studies have associated mutations in *SCN8A* with epilepsy, intellectual disability, and cranial features such as microcephaly (Veeramah et al. 2012; de Kovel et al. 2014; Estacion et al. 2014; Ohba et al. 2014; Vaher et al. 2014; Blanchard et al. 2015; Larsen et al. 2015; Wagnon and Meisler 2015).

## DISCUSSION

Overall, the various analyses employed herein were able to efficiently and accurately detect variants in this family, uncovering a deleterious single-nucleotide variant (SNV) likely contributing to the severe phenotype and previously unidentified in clinical databases such as ClinVar and OMIM. By using existing databases to filter variants by rarity and deleteriousness, common variants and/or benign variants can be excluded from the analysis, leaving a small subset of variants to study and facilitating the analysis. Unlike other tools and protocols currently available, the described protocol efficiently examines the phenotypes associated with the genic location of a mutation by using phenotype analysis tools to efficiently provide a link between a variant and a set of phenotypes. This step prioritizes variants by phenotypic relevance quickly and efficiently, thus eliminating the need to manually research the phenotypes associated with many genes. wANNOVAR outputs were not depicted here because this relies on Phenolyzer to determine relationships to phenotype and was thus similar to the Phenolyzer output.

As in previous studies (Bragg et al. 2013b; Salipante et al. 2014), the Ion Torrent Personal Genome Machine (PGM) sequencing results appeared to have several erroneous calls, especially around homopolymer regions and indels. These errors have been attributed to the PGM “dark sequencing” chemistry, which uses semiconductor chips to measure minute pH differences caused by the release of a hydrogen ion when a nucleotide attaches to a DNA template. In contrast, “light sequencing” platforms such as the Illumina MiSeq and HiSeq use high-resolution cameras or sensors to detect wavelengths of light emitted when a reaction occurs, such as when a fluorescently labeled nucleotide attaches to a prepared DNA template. Even with an optimized variant calling platform, the errors in PGM sequencing can lead to high false-positive rates. It is likely that the high number of de novo mutations, most of which have a low-quality score, were detected by the variant calling pipeline because of the small and sporadic occurrence of sequencing errors in homopolymer regions.

Although several mutations of *SCN8A* have been found to be associated with a similar syndrome (Wagnon and Meisler 2015), there is no entry in ClinVar for the p.Leu267Ser mutation. Interestingly, mutation of the corresponding leucine residue in *SCN1A* resulted in impaired channel inactivation (Kahlig et al. 2008), a common feature of *SCN8A* mutations in epileptic encephalopathy (Wagnon et al. 2016).

Although there are several factors leading to a susceptibility to fractures, including a ketogenic diet often recommended for seizures (Bergqvist et al. 2008), this patient exhibits co-occurrence of a mutation in *SCN8A* and bone fractures.

## METHODS

### DNA Isolation and Sequencing

Genomic DNA was extracted using standard methods (Puregene, QIAGEN). The Life Technologies Ampliseq Exome RDY kit (Thermo Fisher) was used to target the exon regions. Ninety-seven percent of Consensus Coding Sequences, with 5 bp exon padding, were amplified using 294,000 primer pairs. These products were sequenced using the Life Technologies Proton sequencing system with 200-bp reads using a P1V3 chip.

### Variant Calling

The DNA sequence was aligned to the UCSC hg19 reference sequence and variants were called using the Torrent Suite software and the Torrent Variant caller. Only exonic variants and variants at the intron–exon boundary (one or two nucleotides into the intron and one nucleotide into the exon) were reviewed. For each variant considered, the depth of coverage was ≥10× and the quality score was ≥30. Ethnicity and variant frequency were considered during analysis. Analysis of the variants was conducted by two independent reviews using in-house protocols and two commercial software packages, Tute Genomics and Cartagenia v4.1. The variant in the *SCN8A* gene was confirmed by Sanger sequencing, which was completed using standard molecular biological methods as performed at Affiliated Genetics and/or the CSHL Sanger Sequencing Core Facility. American College of Medical Genetics and Genomics (ACMG) reporting criteria were also used to evaluate variants (Richards et al. 2015). The primers used in the Sanger sequencing procedure were as follows:

SCN8A-800T>C—Forward 5′-TTCTGTCTCCTCAGGCCTGA-3′
SCN8A-800T>C—Reverse 5′-TGCAGAGAAGAGGCCACCTA-3′

In additional analyses, binary alignment (BAM) files from the PGM platform were converted to FASTQ files. Variations in the sequenced DNA when compared with a reference genome (variants) were called for each family using the OTG-snpcaller pipeline, which has been reported to have a substantially higher proportion of sequencing reads mapped to a reference genome, lower false-positive rate, and overall lower error rate when analyzing sequences coming from the PGM platform (Zhu et al. 2014). Unlike other sequencing software and pipelines such as the Genome Analysis Toolkit (GATK) and FreeBayes (McKenna et al. 2010; Garrison and Marth 2012), OTG-snpcaller is specifically designed to take into account errors associated with PGM data, such as errors around homopolymers, thus increasing the overall accuracy. Variants were aligned to the hg19 assembly. A VCF file containing information about each mutation was then output (Danecek et al. 2011). The hardcoded OTG-snpcaller pipeline was recoded, without any change to the function of the pipeline, to make it usable for this analysis. Analyses using this pipeline were then completed for the proband, parents, and siblings on a computational cluster located on campus.

### Variant Selection and Prioritization

The resulting VCF file for each individual in each family was then converted into ANNOVAR (avinput) files using ANNOVAR software, as avinput files are easier to analyze because of their simpler format; avinput files provide information regarding chromosome number, start position, end position, reference nucleotide, alternate nucleotide, and quality scores for each variant (Wang et al. 2010). All avinput files for a particular family were then loaded into a Python program, which performs set intersections using DataFrame functions from the Pandas library, and set functions using the Numpy library to identify de novo and autosomal recessive variants (Van Der Walt et al. 2011). Autosomal recessive variants were identified by isolating homozygous variants in the affected child, intersecting these variants with variants that were heterozygous in both parents, and subtracting variants that were homozygous in the siblings. De novo variants were identified by subtracting variants found in the parents and sibling from variants found in the proband, using the following set functions performed using Pandas and Numpy libraries in Python:

$$\begin{array}{rcl} \text{Autosomal recessive} & = & [(M_{\text{het}}\cap F_{\text{het}})\cap P_{\text{hom}}]-{\text{SIB}}_{\text{hom}},\\ \text{de novo} & = & P_{\text{all}}-M_{\text{all}}-F_{\text{all}}-{\text{SIB}}_{\text{all}}\text{,} \end{array}$$

where *M* refers to the mother's variants, *F* refers to father's variants, *P* refers to the proband's variants, and SIB refers to sibling's variants. The subscript het refers to heterozygous variants, hom refers to homozygous variants, and all refers to all variants.

The columns examined included the chromosome number, start point, end point, and zygosity of each called variant. The resulting avinput files were then output as BED files, which contain columns providing chromosome number, start point, and end point of the mutation. This process ensured that the resulting BED files contained all autosomal recessive and de novo variants that could be determined from the VCF.

Using the GATK SelectVariants tool, these two BED files were intersected with the original VCF file two separate times, creating two VCF files, one containing only autosomal recessive variants and one containing only de novo variants. These files were then examined separately for all further analyses.

Both VCF files were then annotated with the Variant Effect Predictor (VEP) software (McLaren et al. 2010), which provided additional information about the variants. This annotated VCF file was then used with Genome Mining (GEMINI) software, which is a powerful, yet flexible network that allows for organization, sorting, and filtering of variants based on VEP and additional annotations. A Structured Query Language (SQL)-type database was created and loaded into GEMINI. After reviewing the GEMINI database schema to examine what annotations could be queried, factors were chosen based on three considerations: rarity, deleteriousness, and read quality. These factors were used as parameters during the GEMINI query to determine which variants would most likely be pathogenic.

Rarity was determined using the ExAC database, which contains population allele frequencies for exonic variants gathered from 60,706 unrelated individuals with no history of “severe pediatric disease” (Exome Aggregation Consortium et al. 2015). Variants were considered rare if not found in ExAC. Deleteriousness was mainly determined by CADD scores, which encompasses 63 annotations to determine a variant's deleteriousness. CADD scores are based on Phred quality scores; therefore, a minimum CADD score of 20, corresponding to the top 1% most deleterious variants, was selected as a cutoff (Kircher et al. 2014). Quality was determined using Phred scores for each variant. Although the resulting quality scores from the OTG-snpcaller pipeline did not correspond to the standard Phred quality score, a minimum cutoff score of 120 was decided after comparing variant calls with their corresponding BAM files. Chromosome number, start point, and end point columns of variants that met these three requirements were queried using GEMINI, and the output was saved as a BED file.

The BED file, along with Human Phenotype Ontology (HPO) terms corresponding to the proband's phenotype, was input into Phenolyzer, which aims to determine and prioritize which mutations contribute most to the phenotype by comparing the provided HPO terms to the phenotypes attributed to the gene in which the proband's mutation is located (Yang et al. 2015). A VCF file containing the same variants as the BED file used with Phenolyzer was then input into similar programs such as wANNOVAR and PhenIX in order to cross-reference several sources (Chang and Wang 2012; Zemojtel et al. 2014).

Additional deleteriousness scores for each variant of interest were also evaluated. SIFT and PolyPhen scores were evaluated for each variant to confirm its predicted deleteriousness. Variants with a SIFT score of <0.05 and a Polyphen score of >0.995 were considered deleterious.

### Confirmation of Variants

Once a possible contributory mutation was identified, its location was then input into GoldenHelix GenomeBrowser, which displayed read information from the BAM files corresponding to each family. All variants of interest were researched thoroughly via PubMed searches and ruled out as major contributing mutations because of no association with a relevant phenotype.

The genic locations of each variant were identified using GEMINI. Initially, the known functions, phenotypes, and diseases associated with each gene were researched using the GeneCards online database, which contains information compiled from more than 100 sources (Rebhan et al. 1998; Stelzer et al. 2011; Dierking and Schmidtke 2014). These results were then confirmed by researching the gene in other databases, such as NCBI, PubMed, and OMIM (Hamosh et al. 2005; Brown et al. 2015). These findings were also compared with the output of the phenotype analysis software. No additional contributing mutations were identified in this individual. The GEMINI query selected no autosomal recessive mutations of interest and 16 rare de novo mutations as mutations of interest. The *SCN8A* mutation was not found in the dbSNP nor in the 1000 Genomes databases.

## ADDITIONAL INFORMATION

### Data Deposition and Access

The sequencing data have been deposited to the NCBI Sequence Read Archive (SRA; http://www.ncbi.nlm.nih.gov/sra) under accession number SRX1815960. The BioSample identifier is SAMN05187971. The mutation in *SCN8A* has been deposited to ClinVar (http://www.ncbi.nlm.nih.gov/clinvar/) under accession number SCV000282046.1.

### Ethics Statement

Research was carried out in compliance with the Federal Policy for the Protection of Human Subjects 45C.F.R.46. The family was recruited to this study at the Utah Foundation for Biomedical Research (UFBR) where extensive clinical evaluation was performed. Written consent was obtained for phenotyping, use of facial photography, and WES through Protocol #100 at the Utah Foundation for Biomedical Research, approved by the Independent Investigational Review Board, Inc.

### Acknowledgments

G.J.L. is supported by funds from the Stanley Institute for Cognitive Genomics at Cold Spring Harbor Laboratory. K.W. is supported by National Institutes of Health (NIH) grant HG006465. M.H.M. is supported by NIH grant R01 NS34509. The authors thank Jacy Wagnon for preparation of Figure 4. The authors acknowledge Jason O'Rawe for bioinformatics support and comments on the manuscript. The authors thank the Exome Aggregation Consortium and the groups that provided exome-variant data for comparison. A full list of contributing groups can be found at http://ExAC.broadinstitute.org/about.

### Competing Interest Statement

G.J.L. serves on advisory boards for GenePeeks, Inc. and Omicia, Inc. and has been a consultant to Good Start Genetics. K.W. is a board member and shareholder of Tute Genomics, Inc. R.R. is an employee, Chief Executive Officer, and shareholder of Tute Genomics, Inc.

## Referees

Anonymous

Keith K. Vaux

## Supplementary Material

Supplemental Material
