# Supplementary material for: SCN8A mutation in a child presenting with seizures and developmental delays
Source: Cold Spring Harb Mol Case Stud. 2016 Nov;2(6):a001073. doi: 10.1101/mcs.a001073 (PMC5111007; doi:10.1101/mcs.a001073)
Supplement: Supplemental Material [file supp_2_6_a001073__index.html]

SCN8A mutation in a child presenting with seizures and developmental delays — Supplemental Material 

# *SCN8A* mutation in a child presenting with seizures and developmental delays

## Supplemental Material

**Files in this Data Supplement:**

- Supp Figures.docx
- Supp VCF files.zip
- Supp Video.mov
- Supp Table 1.xlsx
- Supp Table 2.xlsx
- Supp Table 3.xlsx
- Supp File Descriptions.docx
