# Supplementary material for: SCN8A mutation in a child presenting with seizures and developmental delays
Source: Cold Spring Harb Mol Case Stud. 2016 Nov;2(6):a001073. doi: 10.1101/mcs.a001073 (PMC5111007; doi:10.1101/mcs.a001073)
Supplement: Supplemental Material [file supp_mcs.a001073_Supp_Figures.docx]

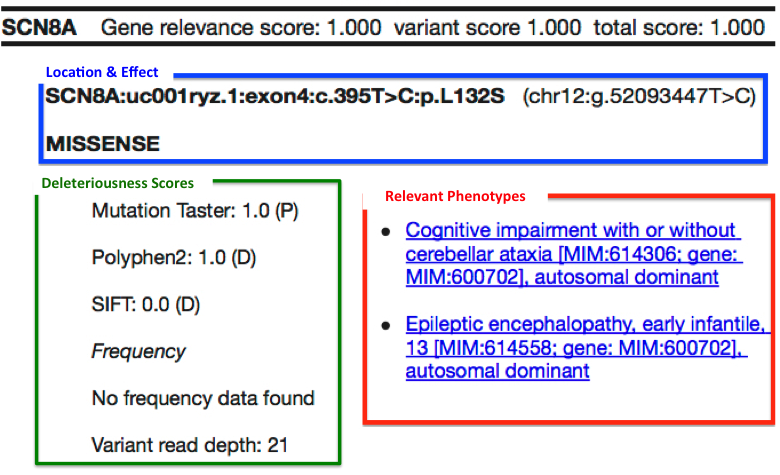


Supplemental Figure 1: PhenIX output for the proband. The mutation in SCN8A was found to be the most likely contributing mutation. PhenIX provides the location, amino acid changes, deleteriousness scores, and relevant phenotypes associated with each variant and each mutation. The mutation in SCN8A was found to be deleterious, and causes epileptic encephalopathy and cognitive impairment, which appear to correspond with the individual’s condition.


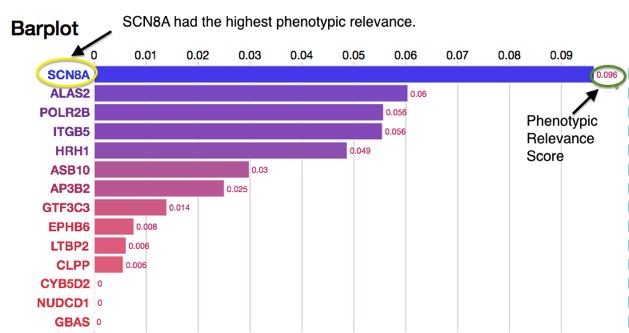


Supplemental Figure 2: Phenolyzer output for the proband. The bar graph indicates that the mutation in SCN8A relates most to the phenotype. It was calculated to have the highest phenotypic relevance score.


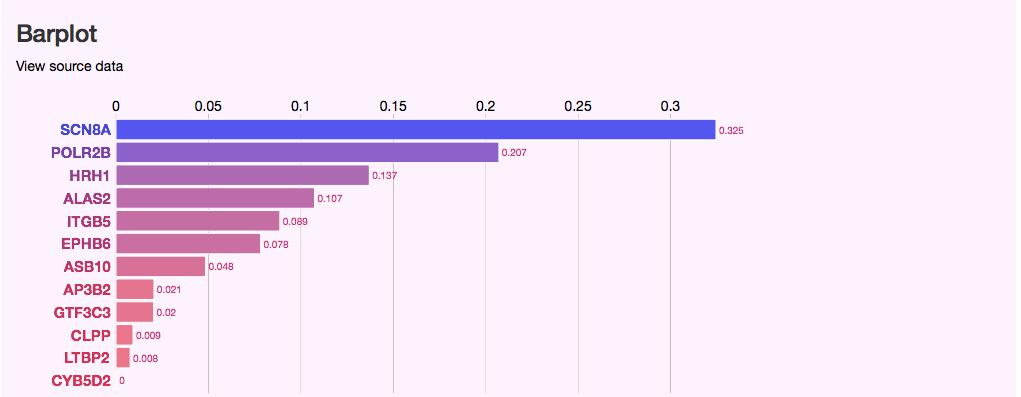


Supplemental Figure 3: wANNOVAR output for the proband. wANNOVAR uses Phenolyzer in order to rank variants by their phenotypic relevance, thus its output looks nearly identical to Phenolyzer’s output.
