# Supplementary material for: SCN8A mutation in a child presenting with seizures and developmental delays
Source: Cold Spring Harb Mol Case Stud. 2016 Nov;2(6):a001073. doi: 10.1101/mcs.a001073 (PMC5111007; doi:10.1101/mcs.a001073)
Supplement: Supplemental Material [file supp_mcs.a001073_Supplementary_File_Descriptions.docx]

**VIDEO DESCRIPTION**

Father describes proband’s seizures usually consist of tonic for 30 seconds, and goes away, or continues with another 30 seconds of tonic/clonic seizure. Then, parents describe how the proband initially met the developmental milestone of shaking a rattle. Proband is able to hold rattle but does not shake it independently. The mother reports she had two large seizures earlier that day so she may not shake the rattle on video. Then, proband is seen in her stroller, with global hypotonia and absent speech. Then, the father of proband describes that prior to being on phenobarbital, the proband would have hundreds of focal seizure per day, sometimes converting into actual seizures.

SUPPLEMENTARY FILES

**1. VCF FILES**

Brother1.vcf

Brother2.vcf

Father.vcf

Mother.vcf

Proband.vcf

Sister.vcf

**EXCEL SPREADSHEETS ILLUSTRATING VAAST RESULTS AMONG THE VARIOUS QUADS**

Supplementary Table 1: Omicia ‘QUAD’ analysis run with Brother 1 designated as the unaffected sibling. VAAST_Quad_K10035_SCN8A_AffectedProbandFemale_And_K10035_SCN8A_unaffectedBrother1 .xlsx

Supplementary Table 2: Omicia ‘QUAD’ analysis run with Brother 2 designated as the unaffected sibling.

Supplementary Table 3: Omicia ‘QUAD’ analysis run with the Sister designated as the unaffected sibling.
